# A breeding method for Ogura CMS restorer line independent of restorer source in *Brassica napus*

**DOI:** 10.3389/fgene.2024.1521277

**Published:** 2025-01-06

**Authors:** Xuesong Wang, Xingyu Liang, Rui Wang, Yuan Gao, Yun Li, Haoran Shi, Wanzhuo Gong, Saira Saleem, Qiong Zou, Lanrong Tao, Zeming Kang, Jin Yang, Qin Yu, Qiaobo Wu, Hailan Liu, Shaohong Fu

**Affiliations:** ^1^ Maize Research Institute of Sichuan Agricultural University, Chengdu, China; ^2^ National Rapeseed Genetic Improvement Center, Chengdu Academy of Agriculture and Forestry Sciences, Chengdu Research Branch, Chengdu, China; ^3^ Oilseeds Research Station, Khanpur, Ayub Agricultural Research Institute, Faisalabad, Pakistan

**Keywords:** *Brassica napus*, double haploid (DH) inducer, Ogura cytoplasmic male sterility, restorer gene, BSA, molecular marker

## Abstract

The Ogura cytoplasmic male sterility (CMS) line of *Brassica napus* has gained significant attention for its use in harnessing heterosis. It remains unaffected by temperature and environment and is thorough and stable. The Ogura cytoplasmic restorer line of *Brassica napus* is derived from the distant hybridization of *Raphanus sativus L.* and *B. napus*, but it carried a large number of radish fragments into *Brassica napus*, because there is no homologous allele of the restorer gene in *B. napus*, transferring it becomes challenging. In this study, the double haploid induction line in *B. napus* was used as the male parent for hybridization with the Ogura CMS of *B. napus.* Surprisingly, fertile plants appeared in the offspring. Further analysis revealed that the cytoplasmic type, ploidy, and chromosome number of the fertile offspring were consistent with the sterile female parent. Moreover, the mitochondrial genome similarity between the fertile offspring and the sterile female parent was 97.7% indicates that the cytoplasm of the two is the same, while the nuclear gene difference between fertile offspring and sterile female parent was only 10.33%, indicates that new genes appeared in the offspring. To further investigate and locate the restorer gene, the BSA method was employed to construct extreme mixed pools. As a result, the restorer gene was mapped to three positions: A09 chromosome 10.99–17.20 Mb, C03 chromosome 5.07–5.34 Mb, and C09 chromosome 18.78–36.60 Mb. The experimental results have proved that induction does produce restorer genes. The induction of the Ogura CMS restorer gene through DH induction line provides a promising new approach for harnessing heterosis in *B. napus*.

## 1 Introduction

Cytoplasmic male sterility (CMS) refers to a maternal genetic trait caused by mutations, rearrangements, or recombination in the mitochondrial genome. In 1968, a scientist named Ogura discovered a type of CMS in the Japanese radish population known as Ogura CMS (Ogura, 1968). This type of sterility is highly stable and unaffected by environmental factors making it an important method to harness the heterosis of *B. napus*. The corresponding restorer gene *Rf* is located in the nuclear genome and inhibits the expression of cytoplasmic male sterility phenotype (Fu et al., 1995). In 1976, the researchers successfully introduced the restorer gene from radish into *B. napus* through intergeneric hybridization (Heyn, 1976). Later, a French researcher Pelletier (Pelletier et al., 1983) utilized the protoplast fusion technology to introduce the restorer gene from European radish into *B. napus* resulting in the selection of Ogura cytoplasmic restorer line of *B. napus*. However, during the transfer of the restorer gene from radish to *B. napus*, fragments of redundant radish genes infiltrated the genome of *B. napus*, leading to issues such as unsatisfactory recovery ability, partial female sterility, and gene linkage between high glucosinolates and the restorer gene. Mitochondrial gene analysis revealed that Ogura CMS is controlled by *orf138* in the mitochondria Yamagishi and Terachi, 1994). Brown identified the restorer gene *Rf* of Ogura CMS and named it *Rfo* (Brown et al., 2003). This gene can alter the post-transcriptional expression level of *orf138* and directly or indirectly reduce the content of *orf138* protein (Desloire et al., 2003; Koizuka et al., 2003). Dahan discovered that *Rfo* encodes a pentapeptide repeat protein consisting of 687 amino acids, referred to as ORF687 (Dahan and Mireau, 2013). Although the sterile gene *orf138* and the restorer gene *Rfo* in the Ogura CMS system have been repeatedly identified (Gaborieau et al., 2016), the exact functional relationship between the two still remains unclear.

In the breeding process, Fu et al. (2018) used artificial breeding combined with chromosome doubling to rapidly select the stable early generation of *B. napus* P3-2. This resulted in the creation of special octoploid *B. napus* new materials (AAAACCCC, 2n = 8x = 76) known as Y3380 and Y3560. These were bred through chromosome doubling after polymerization hybridization between P3-2 and F_1_ hybrid of *B. napus*, F_1_ hybrid of *B. napu* and Chinese cabbage (Luo et al., 2021). When Y3380 or Y3560 was used as the male parent and crossed with normal tetraploid *B. napus*, a large number of offspring similar to the female parent appeared, all of which were homozygous tetraploid. Through various techniques such as field phenotype, flow cytometry, and SNP chip identification, it was confirmed that the phenotype, ploidy, and genotype of the hybrid or test cross offspring were consistent with the female parent. As a result, these materials were named *B. napus* double haploid inducers (DH inducers). The basic principle of induction is that during the induction process the gene of the DH inducer, acting as the male parent, enters the female parent’s egg cell and forms a zygote through fertilization. During zygotic mitosis, the paternal chromosome is specifically lost, and this loss process may cause some genes to penetrate the maternal genome through homologous exchange or transposon hopping. After this, the zygotic haploid genome is doubled and develops into a complete embryo, ultimately forming a double haploid offspring (Zhao et al., 2023). The efficiency of induction by the DH inducer as the male parent varies depending on the female parents. The induction process also has an interaction effect with the genotype and cytoplasmic type of the female parent (Zhang et al., 2021; Zhang et al., 2022). Therefore, by using the DH inducers, the cytoplasmic male sterile lines and restorer lines of *B. napus* can be rapidly and synchronously bred. This allows for the induction of interspecific hybrids of rapeseed and the quick selection of genetically stable offspring (Zhou et al., 2022). Furthermore, the inducers can be used in conjunction with the CRISPR/Cas9 gene editing system to directly modify the genes of rapeseed and cabbage. This allows the production of genetically stable induced offspring without the presence of transgenic components in a single step (Li et al., 2021).

The use of DH inducers to directly pollinate Ogura CMS produces fertile offspring due to cytoplasmic interaction. However, the nuclear gene of the DH inducer was derived from the polymerization hybridization of *B. napus* and *B. rapa*, without the presence of the Ogura CMS restorer gene. Do the induced fertile offspring acquire the restorer gene? To answer this question, this study conducted morphological and cytological observations on the tested materials to determine the phenotype, pollen development, and ploidy of the plants. Molecular markers were utilized to identify the cytoplasmic type and determine whether the indued offspring possessed the restorer gene. Furthermore, mitochondrial genome detection and SNP homozygosity identification were performed on the female parent and its offspring materials. Using the BSA method, the Ogura cytoplasmic restorer gene was initially located, and the allelicity of the restorer gene was identified. These findings provide a new perspective for the breeding of Ogura CMS restorer lines.

## 2 Experimental materials and methods

### 2.1 Experimental materials

The experimental materials used in this study were provided by the Chengdu Academy of Agricultural and Forestry Sciences. The male parent was the octoploid nap cytoplasmic double haploid induction line (DH inducer) Y3560 and Y3380, the female parent was the tetraploid Ogura CMS material (4508A). The tetraploid offspring included plants with the codes 4211, 4213, 4214, 4215, 4216, 4217, 178 and 4630. The positive control was Chuanyou 36 which had the Ogura cytoplasmic restorer gene while the negative control was Zhongshuang 11 (ZS11) which is without a restorer gene. All of the plant materials were grown at the Wenjiang Experimental Base of the Chengdu Academy of Agriculture and Forestry Sciences (Table 1).

**TABLE 1:** The sheet of materials information.

Material category	Material code	Ploidy
experimental group	male parent: Y3560, Y3380 (F_10_ selfing offspring)	octaploid
	female parent: 4508A (Male sterile line)	tetraploid
	induced offspring: 4211, 4212, 4213, 4214, 4215, 4216,4217, 178 and 4630	tetraploid
positive control	Chuanyou 36 (containing restorer gene)	tetraploid
negative control	ZS11 (without restorer gene)	tetraploid

Notes: The F_1_ generation of 4508A × Y3380 was 4211, 4212, 4213, 4214, 4215, 4216 and 4217. 4211 selfing produced fertile offspring 4211C and sterile offspring 4211A. The F_1_ generation of 4508 A × 4211 C was 178, of which 178A was sterile and 178C was fertile. Character separation of 178C selfing F_2_ generation. 4630 is the induced stable offspring containing restorer gene.

### 2.2 Identification of pollen vitality

During the flowering period, fresh buds were collected and anthers were carefully placed on clean slides. Using tweezers the anthers were gently mashed. Next, 1-2 drops of aceto-carmine were added to the mashed anthers. The glass slides were then covered and any excess bubbles were gently pressed with tweezers. Afterward, the excess liquid was wiped away using mirror paper, and finally, the sample was observed under a microscope (Jugulam et al., 2015). If the pollen grains appeared dark red after staining with aceto-carmine, it signifies that the pollen is viable. On the other hand, if the pollen grains retain their original color or the slide remains colorless, it suggests that the pollen grain has lost its vitality or is sterile.

### 2.3 Slice identification of sterile and fertile plants

The buds at various stages were collected and immersed in Carnoy’s fixative (FAA) for 24 h. They were then stored in a refrigerator at −4°C for later use. The buds were categorized based on their length: less than 2 mm, 2–3 mm, 3–4 mm, 4–5 mm, 5–6 mm, and greater than or equal to 6 mm. Buds of different sizes were dehydrated and embedded in Surgipath (Leica) Fsc22 freezing embedding agent, and frozen below −30°C. Sections of approximately 5 nm–10 nm thickness were cut using a Leica_CM1900 freezing microtome. The slides were air-dried, stained with eosin for about 10–20 s, and then examined under a microscope before being stored.

### 2.4 Ploidy detection by flow cytometry

The fresh young leaves were collected, cleaned with 75% alcohol, and dried. A small round leaf with a diameter of 5 mm was then punched out and placed in a clean glass dish. Next, 0.5 mL of pre-cooled LB01 cell lysate was added, and the leaf was quickly shredded with a sharp blade. The resulting mixture was filtered into a 2 mL EP tube using a 35 mm filter head. To this filtrate, 1 mL of PI dye solution was added and gently shaken to mix. The tube was then treated in the dark for 30 min (Crowley et al., 2016; Ureta et al., 2017). The samples were analyzed using flow cytometry (AccuriC6 Plus model) to detect the fluorescence intensity and the number of cells. Approximately 10,000 cells were collected for each sample. Before detection, ZS11 (a tetraploid *Brassica napus*, 2n = 38) was used as a control for ploidy detection (Yin et al., 2020).

### 2.5 Chromosome observation

The preserved materials were removed and washed with deionized water (2–3 times). Then using tweezers, the anthers and stigmas were taken out and placed in a centrifuge tube containing 1 mol/L hydrochloric acid. The material was dissociated in a water bath (at 60°C) for 10–15 min. Once dissociation was complete, the material was gently clamped and washed with deionized water two to three more times, and excess water was absorbed using filter paper. The treated anthers and ovaries were placed on clean slides and gently mashed with separate tweezers (be careful not to use the same tweezer for anthers and ovaries from different materials and remember to clean them with deionized water). Then 2-3 drops of pre-prepared modified phenol fuchsin dye solution, were added and left to stand for 8–10 min for full dying (Li et al., 1995). The cover glass was then placed on top and the excess dye solution was absorbed with filter paper. Using the thumb, the cells were separated from the chromosome by pressing vertically on the coverslip (be careful not to slide the coverslip to avoid overlapping cells and making observations difficult). The samples were placed under the low magnification mirror to locate the image and subsequently observed and photographed under an oil mirror (Yin et al., 2021).

### 2.6 Extraction, purification, and detection of total DNA

The DNA from leaves was extracted using the CTAB method (Doyle, 1991). The extracted genome was then detected using a nucleic acid protein detector. The absorbance 260/280 ratio of the DNA was determined to be between 1.7 and 2.0, indicating good purity. Additionally, the 260/230 ratio was approximately 1.80 suggesting no interference from phenols, polysaccharides, and other impurities). To check the presence of DNA, a 1% agarose gel electrophoresis was performed, and a bright single band greater than 2000 bp was observed. The qualified samples were diluted to approximately 100 ng/μL and stored at −20°C for future use.

### 2.7 SNP purity identification

The first step of this experiment involved extracting DNA from leaf samples. The extracted DNA was then diluted to a concentration range of 50–100 ng/μL. Sample detection: The next set of procedures included whole genome amplification, DNA fragmentation, DNA purification, DNA resuspension, DNA denaturation, DNA hybridization with a chip, single base extension, and staining. Once these steps were completed the chip was scanned and the data was typed. The chip detection was performed by Wuhan Shuanglvyuan Biotechnology Company. The 50K SNP chip is in uniform coverage of 19 pairs chromosomes in *Brassica napus* developed by Wuhan Shuanglvyuan Biotechnology Company, which is based on the Illumina Infinium SNP chip technology. This chip was used for detection, typing analysis, and initial positioning.

### 2.8 Determination of mitochondrial genome

The DNA samples of 4508A and 4211C were sent to Beijing BestMail for second-generation sequencing (Mitochondrial DNA high-throughput sequencing) with a depth of 10X. The data returned by the company were used for SNP and Indel data mining. The SNP data were then used to calculate Nei genetic distance and perform cluster analysis between the samples (McKenna et al., 2010).

Mitochondrial sequence splicing and annotation analysis: The original data returned by the company were spliced into complete mitochondrial genome sequences, a process completed by Wuhan Yiersan Biological Company. Firstly, primers were designed at both ends of the potential contig for amplification and sequencing. The sequences were then compared with the reference genome, to fill any gaps and verify regions with splicing doubt, resulting in the obtainment of complete mtDNA (Tanaka et al., 2012). Repeat sequence analysis, coding gene prediction, non-coding RNA prediction, and genomic component analysis were performed on all samples. Finally, the protein-coding genes, tRNA genes, and rRNA genes of all mtDNA were counted.

### 2.9 Initial mapping of the gene by BSA technique

The F_1_ population was created by crossing 4508A as the female parent with 4211C. The F_1_ population was self-pollinated to obtain F_2_. From the F_2_ population, 20 fertile plants and 20 sterile plants were selected. Young leaves weighing 0.15 g were taken from these plants for DNA extraction. The DNA from 20 fertile F_2_ plants and 20 sterile F_2_ plants were combined in equal amounts to create a mutant mixing pool and a wild-type mixing pool. The initial mapping of the gene used BSA technique by 50K chip developed by Wuhan Shuanglvyuan Biotechnology Company, which is based on the Illumina Infinium SNP chip technology.

## 3 Results

### 3.1 Phenotype and anther section identification of induced offspring

**FIGURE 1:**
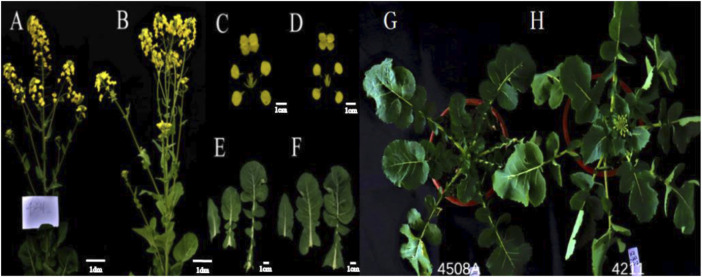
Phenotypic observation of sterile female parent and induced offspring plants. **(A, B)** Plants of 4508A, 4211; **(C, D)** Floral organs of 4508A, 4211; **(E, F)** Leaves of 4508A, 4211; **(G, H)** Young plants of 4508A, 4211.

**FIGURE 2:**
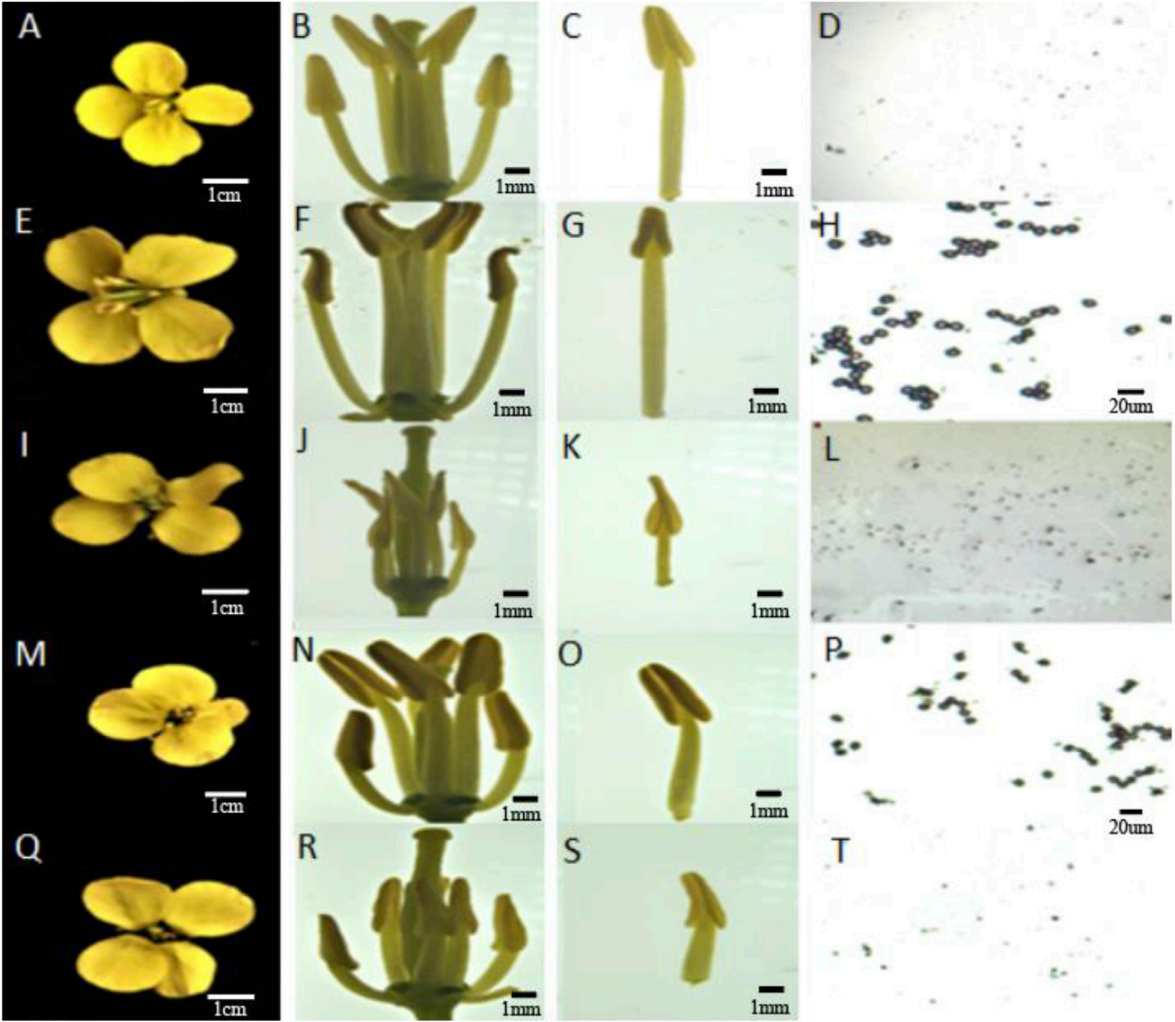
Flower organ and Pollen staining diagrams of sterile female parent and induced offspring. **(A–D)** 4508A; **(E–H)** 4211C; **(I–L)** 4211A; **(M–P)** 178C; **(Q–T)** 178A.

During the flowering period, the sterility rate of the female parent 4508A was determined to be 100%. By pollinating, 4508A with the DH inducer Y3380 as the male inducer (9 of the 125 offspring were fertile), a fertile plant 4211 was obtained (see Figure 1 for phenotypic observation of 4508A and 4211). The plant 4211 then continued to self-pollinate, resulting in the F_2_ generation producing both the fertile plant 4211C and the sterile plant 4211A. Additionally, by crossing 4211C with 4508A the fertile offspring 178C and the sterile offspring 178A were obtained. The sterile plants 4211A and 178A had degraded stamens and elongated, exposed stigmas on the pistils. On the other hand, the fertile plants 4211C and 178C had larger flowers with significantly longer stamens compared to the pistil stigma, and their anthers were filled with pollen. After staining with aceto-carmine, the pollen appeared purple-red (Figure 2). Furthermore, the plant morphology and leaf morphology of 4211C were similar to those of 4508A, while 4508A exhibited self-infertility and 4211C exhibited self-fertility.

**FIGURE 3:**
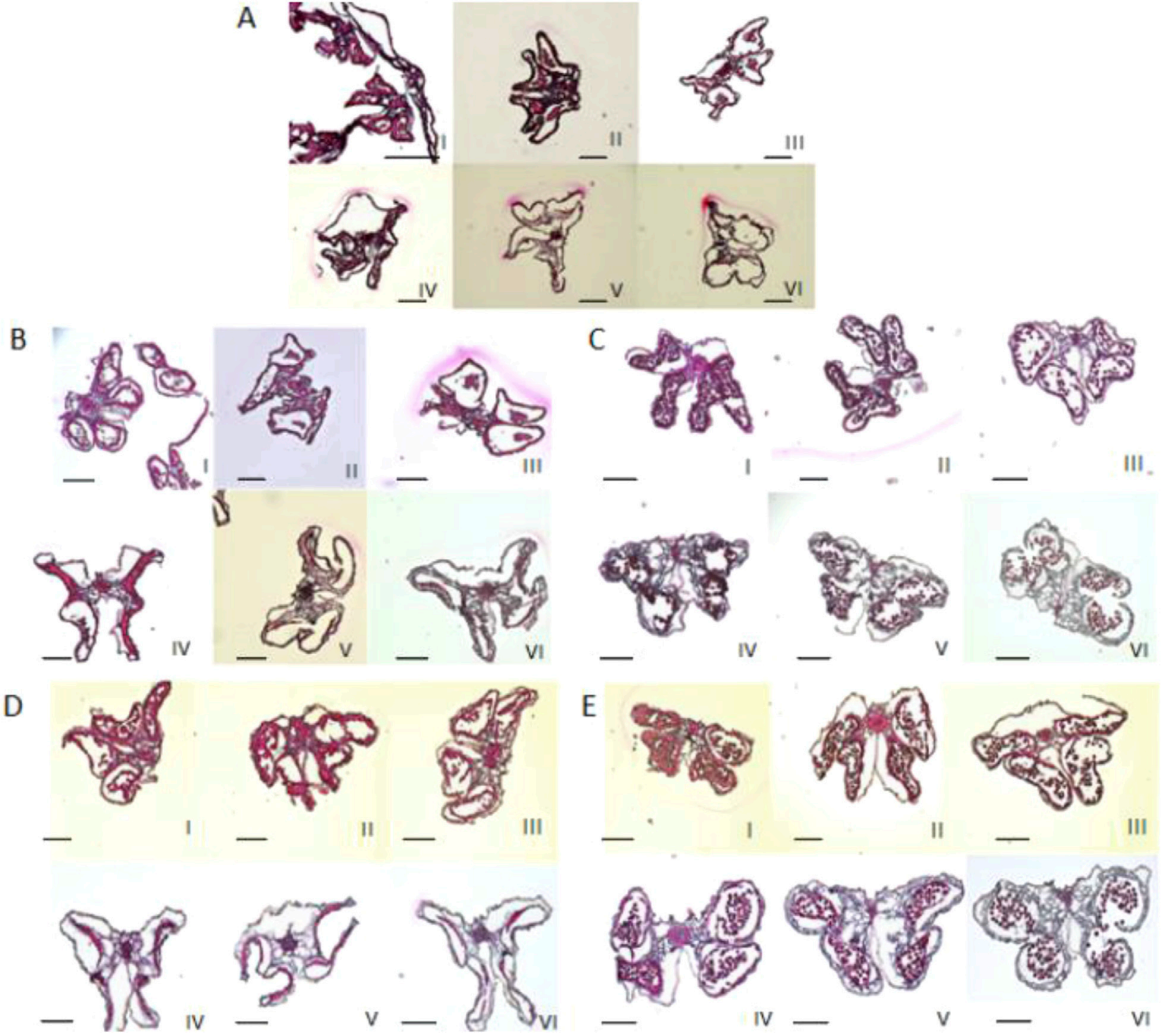
Observation of anther sections at different stages. (I) Less than 2 mm; (II-V) 2–6 mm; (VI) larger than 6 mm. **(A)** 4508A; **(B)** 4211A; **(C)** 4211C; **(D)** 178A; **(E)** 178C. Ruler = 1 mm.

The four pollen sacs of the female parent 4508A and its sterile plant offspring 4211A and 178A were in different stages of abortion. The pollen mother cells of the fertile offspring 4211C and 178C underwent normal meiosis, forming normal tetrads and pollen grains (Figure 3). The female parent 4508A did not have any mature pollen grains in the anther chamber of the pollen sac throughout the entire process, which aligns with the results of pollen viability identification. However, the opposite was observed in offspring 4211A and the female parent. As the anther chamber matured, the tapetum residue gradually decreased, and the pollen sac shrank from its initial full state to a dry state. The analysis suggests that the abortion period may occur in the early stage between the tetrad and the pollen grains.

### 3.2 Ploidy and chromosome number identification of induced offspring

**FIGURE 4:**
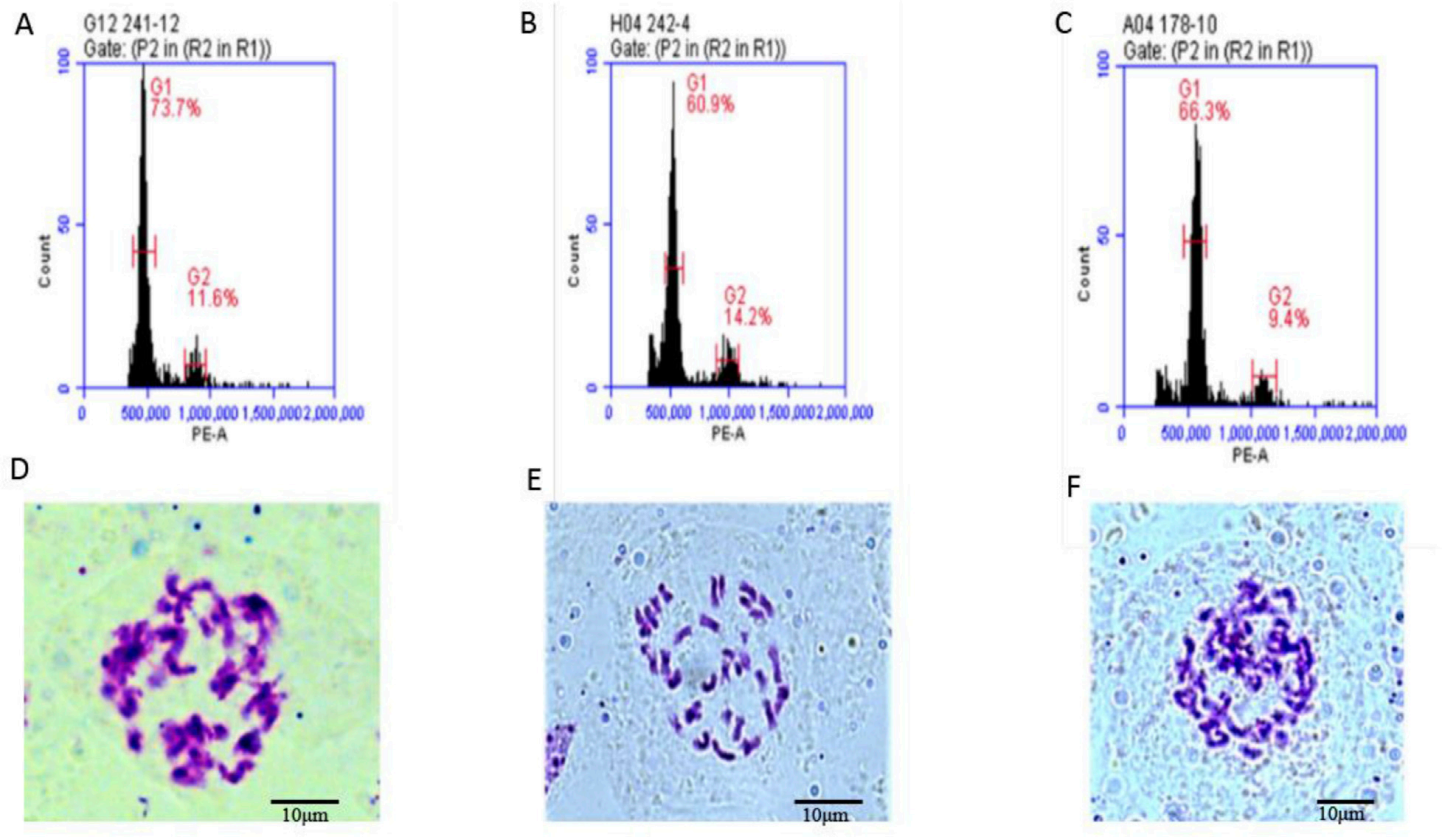
**(A–C)** Flow cytometry ploidy map of 4508A (field code 241), 4211 (field code 242), 178 (4508A and 4211 hybrid F_1_ generation); **(D–F)** Chromosome observation maps of 4508 A, 4211, 178.

To determine, whether the fertile offspring were a result of the male parent or the hybrid of the parents, flow cytometry was used to analyze the ploidy of the parents and the offspring 4211 and 178. The control used was tetraploid *B. napus* Zhongshuang 11 (ZS11) which had a peak of approximately was about 450,000 to 550,000. The results revealed that both the female parent and the offspring had peak values within the same range confirming that they were tetraploid plants (Table 2; Supplementary Figure S1). Additionally, cytological observation, showed that the number of chromosomes in the female parent 4508A and the offspring 4211 and 178 was 38, which matched the number of tetraploid chromosomes in *B. napus* (Figure 4). These findings indicate that the ploidy and chromosome number of the offspring were consistent with those of female parent, rather than being a result of the normal hybridization between the parents.

**TABLE 2:** Flow cytometry peak and ploidy of female parent and induced offspring.

Material	Peak	Plant ploidy	Material	Peak	Plant ploidy	Material	Peak	Plant ploidy
4508A-1	493,182.10	tetraploid	4211-1	504,960.19	tetraploid	178-1	541,941.81	tetraploid
4508A-2	499,763.38	tetraploid	4211-2	470,924.31	tetraploid	178-2	533,351.01	tetraploid
4508A-3	496,333.67	tetraploid	4211-3	526,423.50	tetraploid	178-3	474,846.66	tetraploid
4508A-4	476,639.84	tetraploid	4211-4	484,176.08	tetraploid	178-4	502,470.85	tetraploid
4508A-5	494,143.85	tetraploid	4211-5	523,022.60	tetraploid	178-5	513,437.29	tetraploid
4508A-6	506,747.64	tetraploid	4211-6	483,556.09	tetraploid	178-6	502,829.80	tetraploid
4508A-7	474,492.24	tetraploid	4211-7	527,705.57	tetraploid	178-7	522,605.93	tetraploid
4508A-8	453,988.11	tetraploid	4211-8	494,914.50	tetraploid	178-8	505,619.32	tetraploid
4508A-9	461,213.16	tetraploid	4211-9	494,832.05	tetraploid	178-9	502,994.31	tetraploid
4508A-10	439,380.52	tetraploid	4211-10	497,956.00	tetraploid	178-10	528,531.48	tetraploid
4508A-11	477,780.88	tetraploid	4211-11	519,318.16	tetraploid	178-11	498,480.70	tetraploid
4508A-12	471,759.82	tetraploid	4211-12	468,391.00	tetraploid	178-12	511,720.75	tetraploid
4508A-13	468,842.90	tetraploid	4211-13	475,299.76	tetraploid	178-13	506,961.61	tetraploid
4508A-14	449,507.74	tetraploid	4211-14	451,191.62	tetraploid	178-14	512,357.78	tetraploid
4508A-15	482,869.26	tetraploid	4211-15	466,502.74	tetraploid	178-15	518,013.20	tetraploid
ZS11-1	506,498.80	tetraploid	ZS11-2	517,940.89	tetraploid	ZS11-3	535,824.11	tetraploid

### 3.3 Cytoplasm type, mitochondrial genome, and SNP chip identification results

**FIGURE 5:**
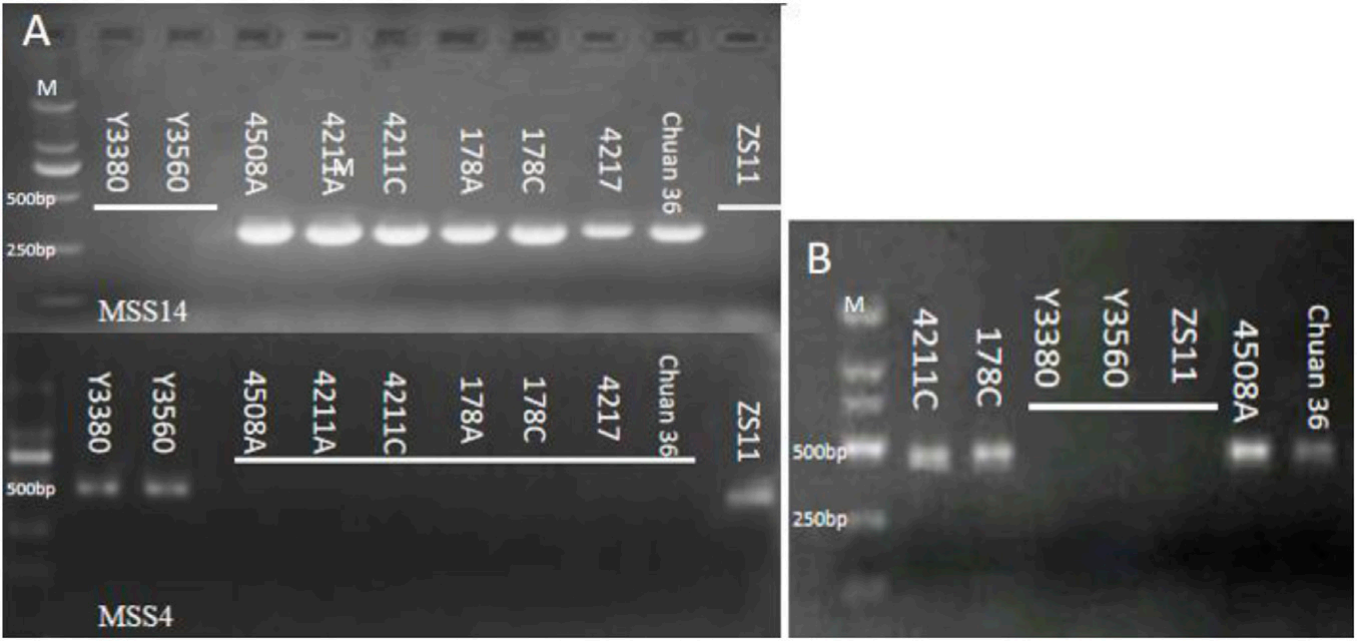
**(A)** Electrophoretogram of cytoplasm type identification; **(B)** Electrophoretogram of gene *orf138*.

To access the cytoplasmic changes pre- and post-induction, nap CMS molecular markers MSS-2, MSS-4, and Ogura CMS molecular markers MSS-13, MSS-14, or MSS-21 (Supplementary Table S1) were utilized for identification. The findings revealed that the DH inducers Y3560 and Y3380 possessed nap cytoplasm, while the female parent 4508A and the induced offspring had Ogura cytoplasm (Figure 5A).

To investigate any alterations in mitochondria pre- and post-induction, the mitochondrial genomes of the female parent and the induced offspring were sequenced and assembled (Supplementary Figures S2A, B). The results indicated that the mitochondrial genome length of 4508A was 280.481 bp and that of 4211C was 280.795 bp. The GC content amounted to 50.5%, and the similarity between the two genomes was as high as 97.7%. The number of lines depicted in the linear alignment diagram matched the number of lines obtained by BLAST, confirming that the mitochondrial genome remained largely unchanged before and after induction (Supplementary Figure S2C). In addition, the gene *orf138* related to sterility of Ogura cytoplasm in mitochondria was cloned in the induced fertile offspring. (Figure 5B, Sequence alignment in Supplementary Figure S3).

**FIGURE 6:**
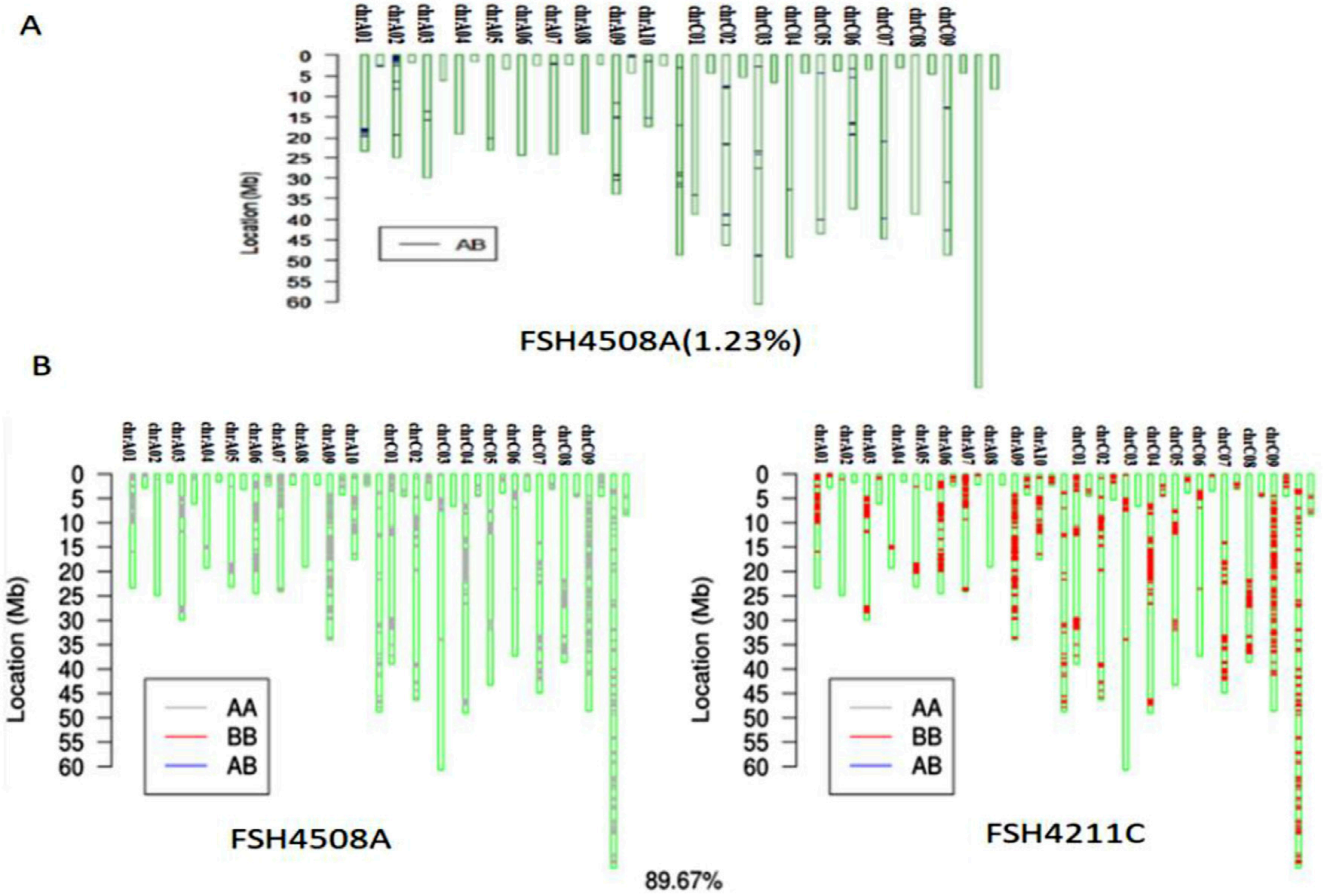
**(A)** Identification map of SNP homozygosity of 4508A; **(B)** SNP comparison between 4508A and 4211.

To further clarify the genetic relationship between the induced offspring and parents as well as the changes within the gene, a SNP chip identification was conducted. The genetic similarity between 4211C and the induced paternal parent Y3380 was 50.67% (Supplementary Figure S4). The heterozygosity of 4508A was 1.23% (Figure 6A), indicating a high level of homozygosity. After excluding the heterozygous region of 4508A, the genetic similarity between the induced offspring 4211C and the female parent 4508A was determined to be 89.67% (Figure 6B).

The cytoplasm type of the offspring remains the same as that of the female parent (Ogura cytoplasm), and no significant changes were observed in the mitochondrial genome. However, the similarity in SNP homozygous identification between the two is close to 90%, suggesting that the male parent primarily plays an inducing role.

### 3.4 Molecular marker identification of restorer genes in induced offspring

**FIGURE 7:**
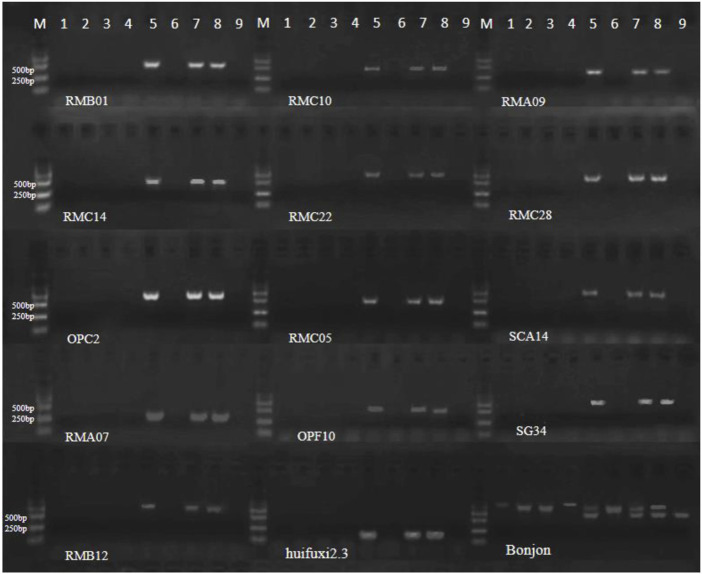
Electrophoretogram of molecular markers of restorer lines. 1–9 represent Y3380, Y3560, 4508A, 4211A, 4211C, 178A, 178C, Chuanyou 36 and ZS11 (Bonjon, there are two bands in restorer lines).

To determine the presence of the Ogura cytoplasmic restorer gene in the induced fertile offspring, we used 28 molecular markers (Hu et al., 2008; Primard-Brisset et al., 2005; Zhang et al., 2013) specific to the large fragment locus of the restorer gene (Supplementary Table S2). The restorer line Chuanyou 36 was used as a positive control, while the nap cytoplasmic ZS11 was used as a negative control. Out of the 28 molecular markers, 15 pairs showed consistent results with the control group (Figure 7; Supplementary Figure S5). The male parent DH inducers Y3560 and Y3380, the female parent 4508A, the sterile plant offspring 4211A and 178A and negative control ZS11 did not exhibit any band. This indicates the absence of the restorer gene fragment. On the other hand, the fertile offspring 4211C, 178C, and the positive control Chuanyou 36 displayed specific bands, indicating the presence of the restorer gene in the fertile plants of the offspring. Only 15 pairs of primers indicated that there was a shortened *Raphanus* sativus fragment and different from the reported *Brassica napus* recovery materials (Most of the restorer genes of *Brassica napus* restorer lines were derived from *Raphanus* sativus Ogura restorer lines, and a large number of linked *Raphanus* fragments were carried at the same time).

### 3.5 Initial localization of the restorer gene by BSA

**FIGURE 8:**
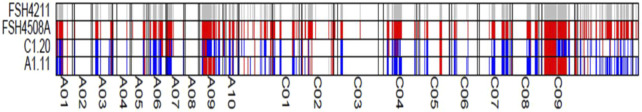
BSA identification results diagram. FSH4211, the male parent 4211C genotype of F_1_; FSH4508A, the female parent 4508A genotype of F_1_; C1.20, the fertile genotype of F_2_ generation; A1.11, the sterile genotype of F_2_ generation. (blue indicates heterozygous genotype, white indicates homozygous genotype consistent with FSH4508A, and red indicates homozygous genotype different from FSH4508A).

The restorer genes were initially mapped using a 50k SNP chip combined with the BSA technique (Plants of F_2_ generation: 128 were fertile and 25 were sterile). Three segments related to the restorer gene were identified on the A09 chromosome (10.99–17.20 Mb), the C03 chromosome (5.07–5.34 Mb) and the C09 chromosome (18.78–36.60 Mb) (Figure 8).

To verify the accuracy of mapping results, 3 pairs of molecular markers (Supplementary Table S3) were designed in the initial location segments to detect the fertile and sterile plants in the F_2_ generation. The results of Bn09-1 (chrC09: 19590617–19590251; chrA09: 12956843–12956567), Bn09-2 (chrC09: 19086365–19086611), and Bn09-3 (chrC09: 19788489–19788380; chrA09: 13137389–13137280) were completely consistent with the field observation indicating the reliability of the initial positions. Additionally, the sequence alignment results obtained from the fertile plants in the F_2_ generation were consistent (Supplementary Figure S6; Supplementary Table S4).

### 3.6 Allelic verification of restorer genes

The stable Ogura cytoplasmic restorer line Chuanyou 36 was the female parent for hybridization. The induced fertile plants namely, 4211, 4212, 4213, 4214, 4215, 4216, and 4217 were used as the male parent. The F_2_ generation of 4212 and 4213 exhibited complete fertility, indicating that their restorer genes were completely allelic to the Chuanyou 36. However, the fertility of the F_2_ generation of 4211, 4214, 4216, and 4217 showed segregation. The restorer genes of 4214 and 4216 were not completely allelic to Chuanyou 36. The fertility segregation existed in 4211 and 4217, resulted in a larger segregation ratio in the F_2_ generation, suggesting allelic variation in the induced restorer genes (Table 3).

**TABLE 3:** Fertility statistics table of allelic identification.

Number	Female parent	Male parent	Paternal fertility	F_2_ fertile number	F_2_ sterile number	Total number	Fertility rate	Sterility rate
1	Chuanyou 36	4211	−	37	13	50	74%	26%
2		4212	+	13	0	13	100%	—
3		4213	+	62	0	62	100%	—
4		4214	+	38	3	41	92.7%	7.3%
5		4215	+	—	—	—	—	—
6		4216	+	59	5	64	92.2%	7.8%
7		4217	−	20	2	22	90.9%	9.1%

Notes: (+) fully fertile, (−) fertility separation.

## 4 Discussion

DH inducers of the male parent *Brassica napus* are new octoploid rapeseed materials with a special ability to induce, which are rapidly selected through artificial synthesis combined with chromosome doubling (Fu et al., 2018). The study found that there are differences in the induction ability of the paternal parent among different maternal parents. When the other Ogura CMS materia was induced, there were occurrences of male parent gene fragment infiltration and hybridization, and purple leaf plants appeared in the F_1_ generation induced by parents without purple leaves (Zhang et al., 2021). In most species, tapetal cells undergo programmed cell death before flowering. The timing of tapetal rupture is crucial for pollen viability, and premature degradation or delay can result in pollen abortion (Kawanabe et al., 2006). Studies have shown that anther abortion of Ogura CMS occurs during the tetrad development stage (González-Melendi et al., 2008; Yang et al., 2008). In the current study, the tapetum of the sterile anther 4508A disintegrated earlier, but the entire process was complete. This suggests that the abortion period of 4508A may occur after the tetrad stage.

The gene *orf138* in mitochondria is associated with infertility traits (Liu et al., 2016; Bonhomme et al., 1992; Landgren et al., 1996; Heng et al., 2014; Jing et al., 2012). By sequencing and assembling the mitochondria of the female parent 4508A and the fertile offspring 4211C, we found that their sequence arrangement was consistent and their similarity was as high as 97.7%. This supports the conclusion that the CMS traits are maternally inherited and that rearrangement of mitochondrial DNA can lead to the failure to produce normal pollen (Shull, 1980; Horn et al., 2014). In terms of cytoplasmic type, ploidy, and chromosome number the inducer material used as the male parent is the octoploid nap cytoplasm, while the induced offspring have the same tetraploid Ogura cytoplasm and 38 chromosomes as the male sterile female parent. In SNP chip detection, the genetic similarity between the fertile plant 4211C and the female parent 4508A was 89.67%, indicating that the genetic material of the nuclear gene mainly originated from the female parent. At the same time, in 10.33% differences, new genes different from the female parent were produced, and the above experiments confirmed that they contained restorer genes. Analysis of the parental materials revealed that there was no homologous sequence linked to the restorer gene. In terms of allelic identification, 4211, 4212, 4213, 4214, 4215, 4216 and 4217 were the F_1_ generation of Y3380 and Ogura CMS 4508A, and the fertility segregation appeared in the F_2_ generation of 4211 and 4217, indicating that the restorer gene was not homozygous. When 4211 and 4217 were crossed with chuanyou36 (a stable Ogura CMS restorer line material), because they were not completely allelic with the recovery gene of chuanyou36, the fertility segregation ratio of the offspring was larger. The selfing F_2_ generations of 4212, 4213, 4214, 4215 and 4216 were completely fertile, indicating that the restorer gene was homozygous. When they were completely allelic to the restorer gene of chuanyou36, the hybrid offspring were 100% fertile, when they were not completely allelic, the offspring will show fertility segregation. It also indicates that there is more than one type of induced restorer gene, and they are not completely allelic to the normal Ogura CMS restorer gene.

Further tests were conducted by crossing 4211C with the original sterile plant 4508A, and the offspring still showed fertile plants (178C). This fully demonstrated that the restorer gene was induced in the offspring plants. BSA was used to localize the induced restorer gene. The results showed that it was located on the A09 chromosome: 10.99–17.20 Mb, C03 chromosome: 5.07–5.34 Mb, and C09 chromosome: 18.78–36.60 Mb, which was consistent with the previous studies on the localization of the restorer gene on C09 (Nie et al., 2016; Uyttewaal et al., 2008). However, through the cloning and sequencing of the restorer gene fragment, it was observed that the induced restorer gene was not the same as the existing restorer lines with the report in past. Further study is needed to understand the mechanism of induced restorer genes when there is no restorer source in parents.

The residual of radish segments leads to poorer agronomic traits in offspring. This study will first verify whether the source of the recovery gene is related to the residual radish segments, and then localize the position of the gene on the chromosome using fluorescence *in situ* hybridization technology to determine if it is random or fixed. Next, we will further explore the regularity of specific chromosomal variations induced by DH inducers, so as to control the behavior of chromosome variation and shorten the remaining fragments of radish.

## 5 Conclusion

The offspring’s high similarity to the maternal genome has been confirmed through phenotype, cytological observation, mitochondrial genome, and SNP chip identification. Instead of the normal hybridization of the parents, the male parent played a role in the induction of double haploid. During the induction process, the restorer gene of Ogura CMS did appear but the experiment showed that it has allelic differences with the existing restorer genes. The Ogura CMS line was directly pollinated by the DH inducer, allowing for the quick breeding of the restorer line without relying on the restorer source. This study introduces a new approach to harness the heterosis of Ogura cytoplasmic male sterile lines in rapeseed and provides a new breeding method to expedite the creation of a three-line method for *Brassica napus.* The mechanism or principle behind the induction of the CMS restorer gene still requires further study.

## Data Availability

The original contributions presented in the study are included in the article/Supplementary Material, further inquiries can be directed to the corresponding authors.

## Abbreviations

CMS, Cytoplasmic male sterile; DH, Doubled haploid.
