# Supplementary material for: A breeding method for Ogura CMS restorer line independent of restorer source in Brassica napus
Source: Front Genet. 2025 Jan 6;15:1521277. doi: 10.3389/fgene.2024.1521277 (PMC11743515; doi:10.3389/fgene.2024.1521277)
Supplement: Supplementary file 1 [file DataSheet1.pdf]

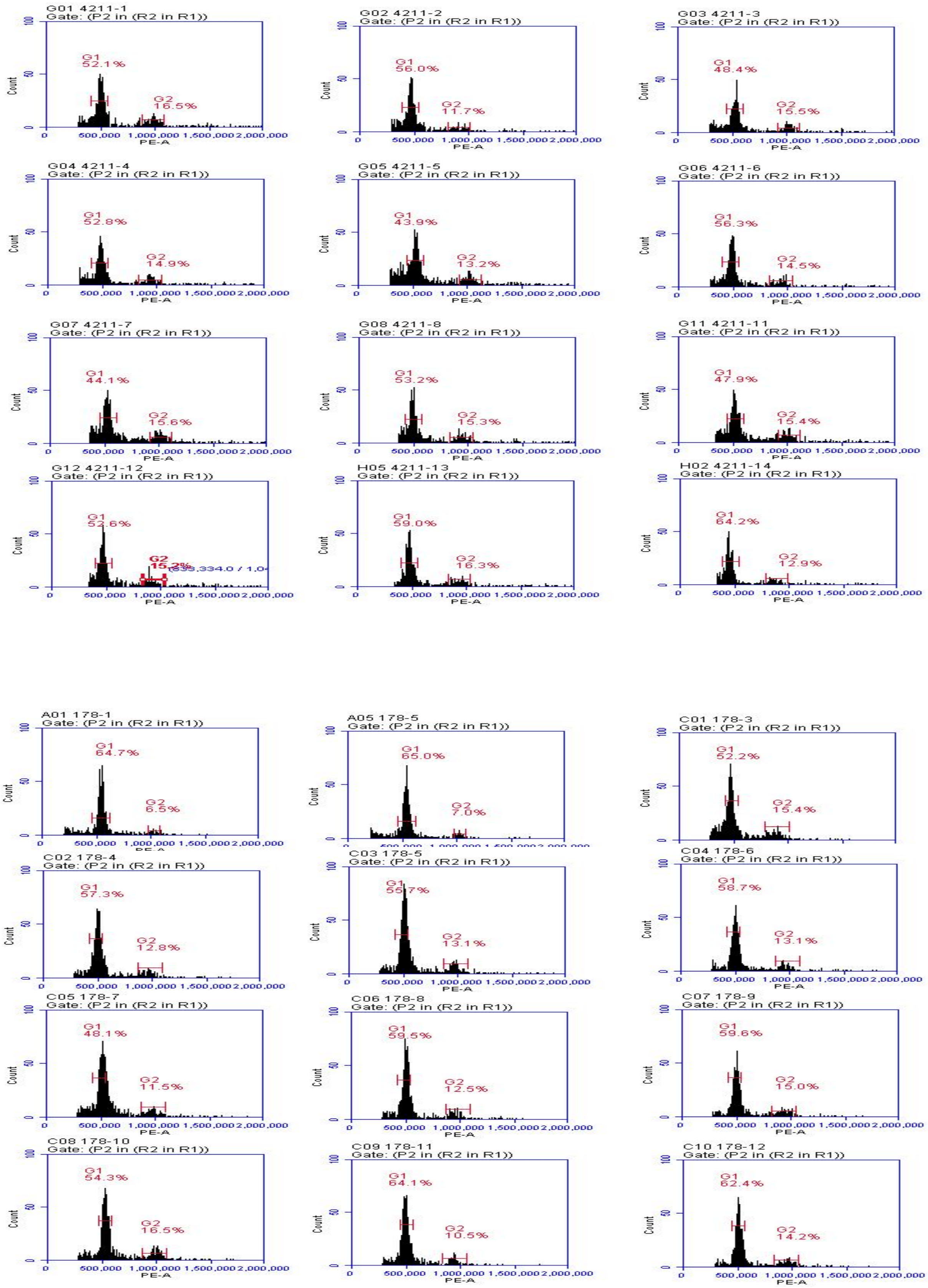

Supplementary Figure 1 Flow cytometry ploidy maps of part of induced offspring 4211 and 178.

Supplementary Table 1 Cytoplasmic primer sequences.

Name	Forward primer (5'-3')	Reverse primer (5'-3')
MSS-2	GAAGTTGAAAAAGGGAATGT	GATCAAGACGAGGAGTAGCT
MSS-4	AGTATTTCGTTC ACCTTGGC	GCTTGGTGGAAAAGATCGTA
MSS-13	TAGCGCTATCTTTCGGCCCT	ACCCGA AGGTCCTGGTCTCT
MSS-14	GCTCGTTCGATTAAGCTCAA	GAATTCCTCTTTCATTGCGG
MSS-21	GGATTATCCTGTGGTGTGGC	CACATGGTGGGTTTGTTGGA

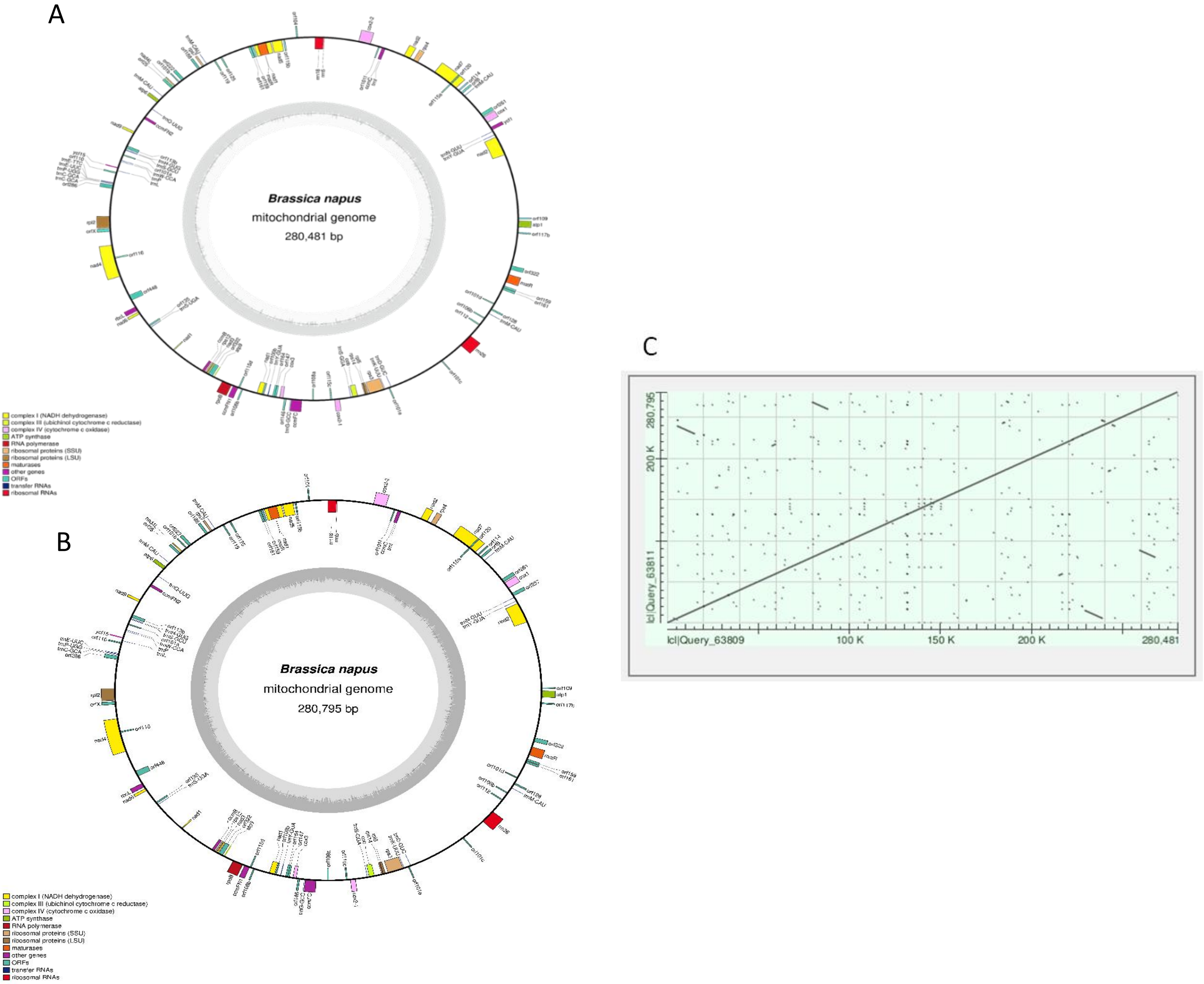

Supplementary Figure 2 **(A)** Mitochondrial assembly annotation diagram of 4508A; **(B)** Mitochondrial assembly annotation diagram of 4211; **(C)** Linear comparison of 4508A and 4211C mitochondrial genomes.

```
4211C.seq           ........................................     40
178C.seq            ........................................     40
chuanyou36.seq      ........................................     40
NCBI-orf138.seq     ........................................     40
Consensus           atgattaccttttttcgaaaaattgtccactttttgtcata

4211C.seq           ........................................     80
178C.seq            ........................................     80
chuanyou36.seq      ........................................     80
NCBI-orf138.seq     ........................................     80
Consensus           atctcactcctactgaatgtaaagttagtgtaataagttt

4211C.seq           ........................................    120
178C.seq            ........................................    120
chuanyou36.seq      ........................................    120
NCBI-orf138.seq     ........................................    120
Consensus           ctttcttttagctttttttactaatggcccatatttggcta

4211C.seq           ........................................    160
178C.seq            ........................................    160
chuanyou36.seq      ........................................    160
NCBI-orf138.seq     ........................................    160
Consensus           agctggttttctaacaaccaacattgtttacgaaccatga

4211C.seq           ........................................    200
178C.seq            ........................................    200
chuanyou36.seq      ........................................    200
NCBI-orf138.seq     ........................................    200
Consensus           gacatctagagaagttaaaaattccatatgaatttcagta

4211C.seq           ........................................    240
178C.seq            ........................................    240
chuanyou36.seq      ........................................    240
NCBI-orf138.seq     ........................................    240
Consensus           tgggtggctaggtgtcaaaattacaataaaatcaaatgta

4211C.seq           ........................................    280
178C.seq            ........................................    280
chuanyou36.seq      ........................................    280
NCBI-orf138.seq     ........................................    280
Consensus           cctaacgatgaagtgacgaaaaaagtctcacctatcatta

4211C.seq           ........................................    320
178C.seq            ........................................    320
chuanyou36.seq      ........................................    320
NCBI-orf138.seq     ........................................    320
Consensus           aaggggaaatagaggggaaagaggaaaaaaaagaggggaa

4211C.seq           ........................................    360
178C.seq            ........................................    360
chuanyou36.seq      ........................................    360
NCBI-orf138.seq     ........................................    360
Consensus           aggggaaatagaggggaaagaggaaaaaaaagaggggaaa

4211C.seq           .............................AGA.G......    400
178C.seq            .............................AGA.G......    400
chuanyou36.seq      .............................AGA.G......    400
NCBI-orf138.seq     .............................GAG.T......    400
Consensus           ggggaaatagaggggaaagaggaaaaaaa   g tggaaa

4211C.seq           ................                            414
178C.seq            ..............AA                            416
chuanyou36.seq      ..............A.                            415
NCBI-orf138.seq     ..............TA                            416
Consensus           atggaccgagaaaa
```

Supplementary Figure 3 Sequence alignment of *orf138* in induced fertile offspring.

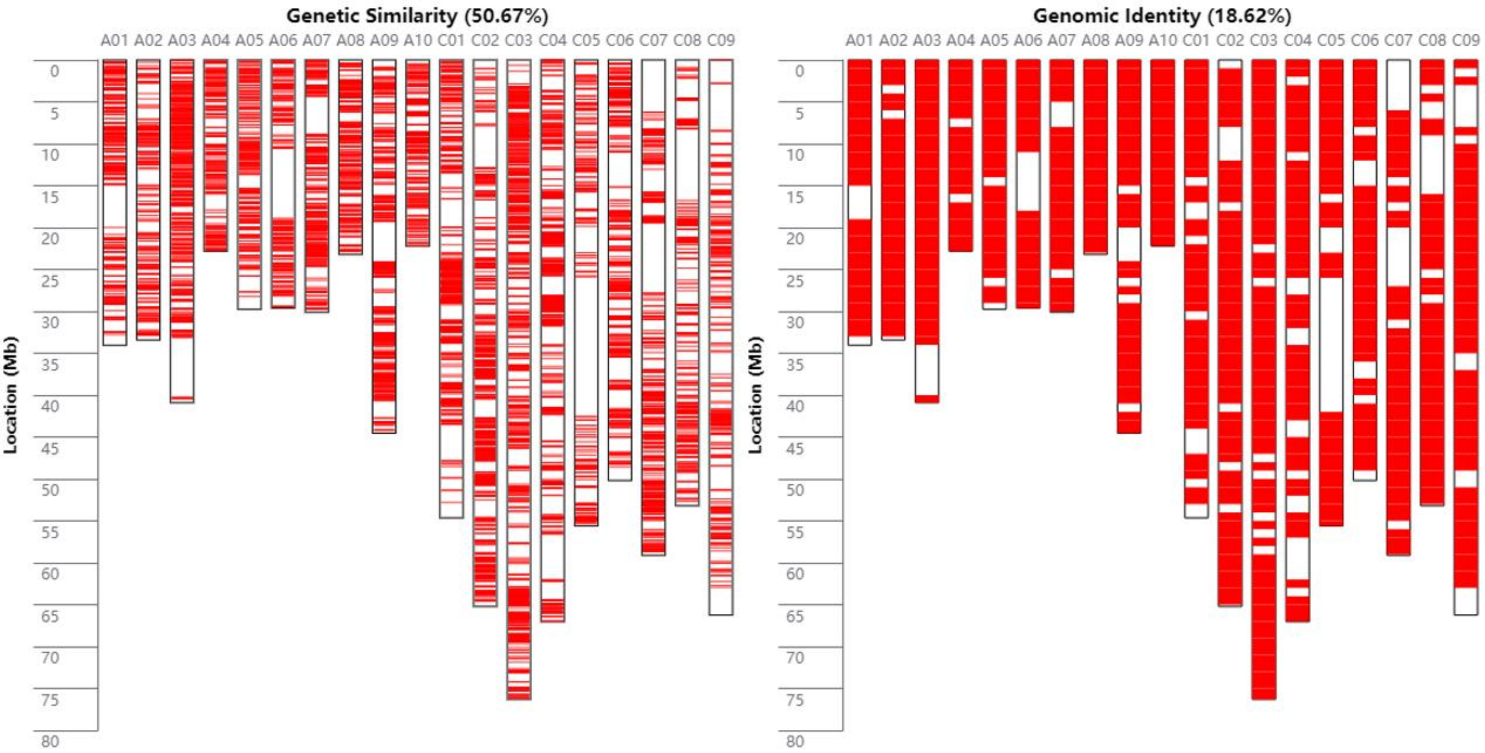

Supplementary Figure 4： Genetic similarity was 50.67% (left) and genomic identity was 18.62% (right) between 4211C and its paternal parent Y3380. (The genome identity: The rapeseed genome was divided into 969 bins according to 1MB as a section. If there was a SNP difference in this bin, it was considered that the two samples were different in this section, which avoided the error caused by the discrete difference between the two samples in the whole genome and the high genetic similarity.)

Genetic similarity = (total number of loci-difference number of loci) / total number of loci x100 %

Genomic Identity = (total bin number-difference bin number) / total bin number x100 %

Supplementary Table 2 Sequence of molecular markers of restorer lines.

Name	Forward primer (5'-3')	Reverse primer (5'-3')
BoIJon	GATCCGATTCTTCTCCTGTTG	GCCTACTCCTCAAATCACTCT
OPF10	AACTTTTTGTGTTTGATTTCTTGC	ACTCCTTCTAAACAAAACCAAACA
OPF20	CCTTAGTTTAGTTGTAGGTGGTGG	AGAAACCGCTCAATTTTAACATAA
RMA05	AAGCTCAGGCTCCTTCACCG	GGGAAGGAGATCCGGACTCA
RMA07	CGGACTCTTTAGCTCCGCCA	CACCTCCTGTCGGCATCTCA
RMA09	TTTTTCAATGCTTCTGTGCAG	GCACAAAATTACAATCAGCGC
RMB01	ATTGTCGTTGTCGATGCATC	AGAAGAAGAAAGTGCCAAGCA
OPH03	TCCACTCCTAGTTCACAATCTATTTT	TATACAAAATGTTGGAATACACAAGG
RME01	TCCATTGCAGAATTCACCTG	TGTTTTCTTCGTCATGTCGG
RME04	GGTCTCAGGTTTTGTGGGAG	GGTTCTCAAAGATTCCGAGG
RMC17	TTTCCACACAAATCGGATTTAA	TGGCCAATGAAAGTTTACTGAT
RMC22	GCTCTACGAGTGAGGATCAAAG	CACTTTCGGAATCCAAGCTC
OPC02	CTGTAACTTTCAACCCAACTCGTAGAA	TTTTGGGGATTACTCTTCTTAGCTTTC
RMC28	AGACCAAGAGGAAGCGTAGC	AAGAAACAACCCAGACTCCG
RMB12	TGGACTAAGAAAGGGTCAGGTA	CGAAGAATCTCTACTCTGTTGT
RMC05	TGCGAGTTTAATCCGGACGC	CCGCGTTATTCTGGTTCAGAGA
RMC08	GAGGCGAAAACATAAACAAGG	ATCGCCAAAACTGTTTCAGG
RMC10	TCCTGCAGTTTGAAATCCTTG	AAGTTTCCCCAAACCAACTTC
RMC14	TTGACGGTTACCCAAAATACCG	TTGATTGCTTCACCCTCACCC
huifuxi23	GGCTAGGGTTTGTGGATTCAA	CAAACTCACTCCTCCAAAACCT
huifuxi45	TGGTCAACTCATCAGGCTC	GCCTCTAGGAGTAGTGAAGAAC
ScA14	TCTGTGCTGGTGGGATGAAAG	TCTGTGCTGGCTACTTATTCATACAG
ScH03	AGACGTCCACCATACC	AGACGTCCACTCCTAGTTC
SG34	TATATTGTACCTTTGCCTCTTC	CTTTTCTTTTAGTTTTTGGTTT
PGIint	CAGCACTAATCTTGCGGTATG	CAATAACCCTAAAAGCACCTG
PGIol	TCATTTGATTGTTGCGCCTG	TGTACATCAGACCCGGTAGAAAA
CP148	AATTTCTCCATCACAAGGACC	CTTCATTGACATCGGAAACTTCT
BnRfo-AS2	CATGCTTCGATCTCGTCCTTTA	GGTAACAACATCAGGGTGGAGT

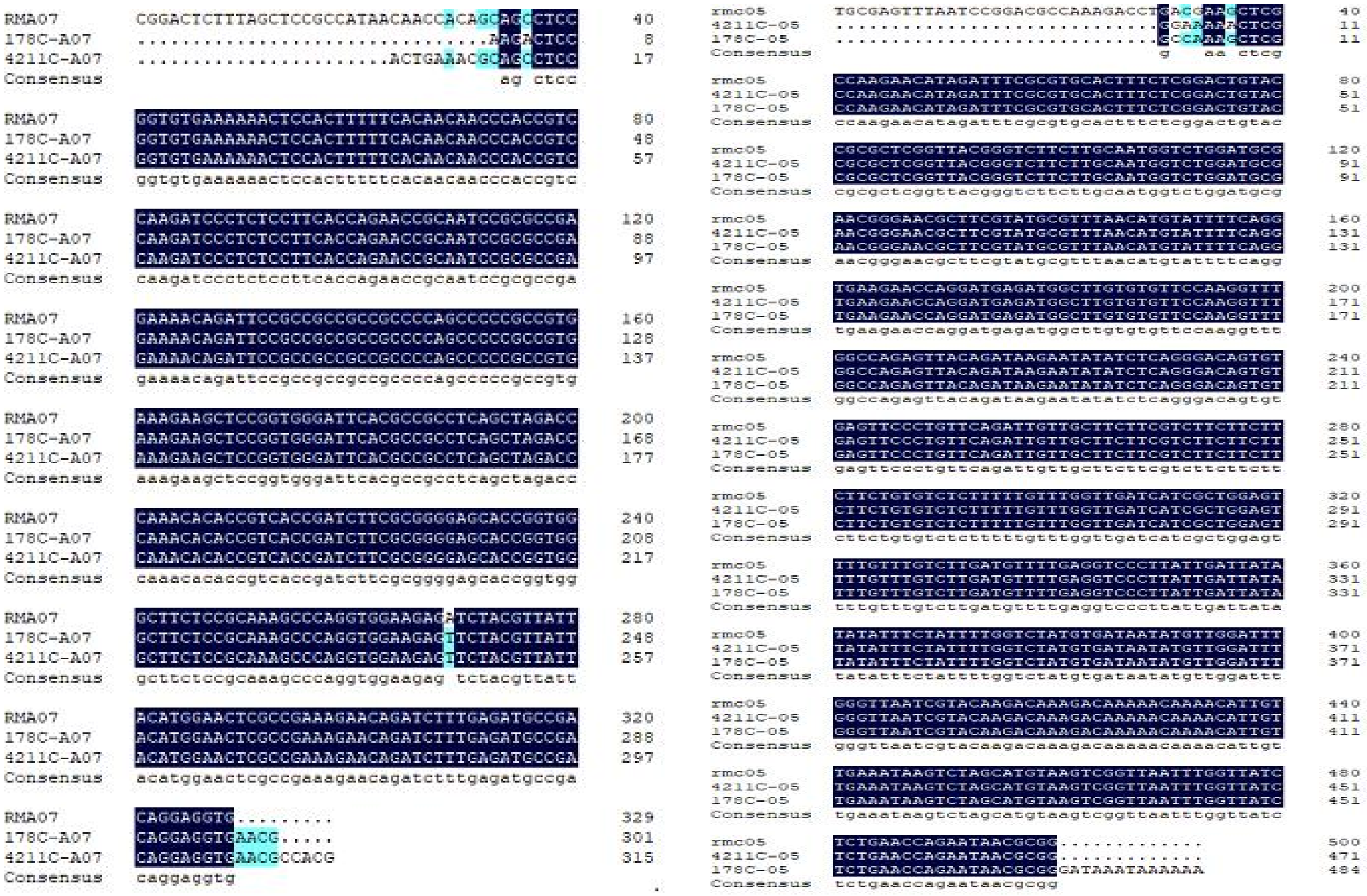

Supplementary Figure 5 RMA07, RMC05 molecular marker sequencing alignment. In the figure, RMA07, RMC05 represent the original sequence of primers; 4211C and 178C represent the sequences amplified by primers in plants, respectively (display part of the molecular marker amplified sequence).

Supplementary Table 3 Primer sequence table.

Name	Forward primer (5'-3')	Reverse primer (5'-3')
Bn09-1	CATTTGGTTTGTCCGTGTGT	AGGGCGACAACCTCTTTCAAC
Bn09-2	TCACATTAGTAAAACGATTGTCCAC	GATTGATTTTTTGGACTCCGTT
Bn09-3	AATAGAGGGAGAGGATGAAAGAAC	AGCTACCTAACAGGTTTTGTTATAAAG

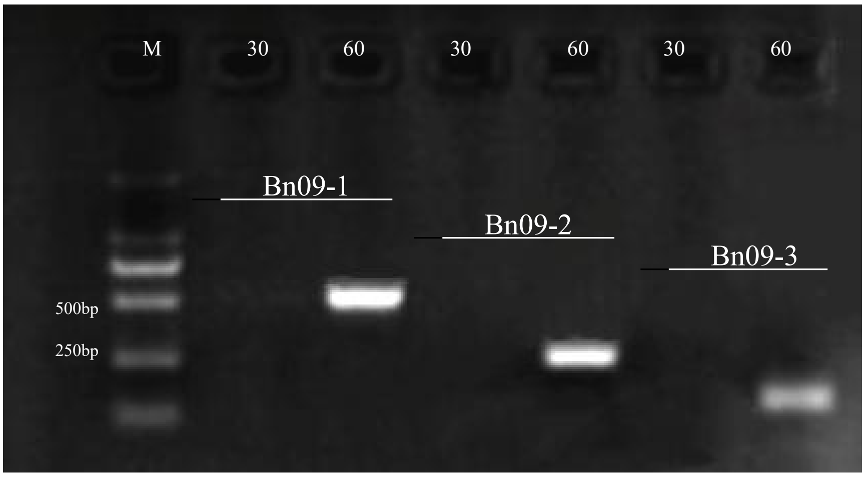

Supplementary Figure 6 Molecular marker electrophoresis map of F_2 generation. 30, 60 represent materials 4630-30, 4630-60 (part of the plant materials were displayed).

Supplementary Table 4 Statistical table of molecular marker results of F_2 generation.

Serial number	Field code	Field test results	Bn09-1	Bn09-2	Bn09-3	Serial number	Field code	Field test results	Bn09-1	Bn09-2	Bn09-3
1	4630-1	F	+	+	+	31	4630-31	S	-	-	-
2	4630-2	F	+	+	+	32	4630-32	F	+	+	+
3	4630-3	S	-	-	-	33	4630-33	F	+	+	+
4	4630-4	S	-	-	-	34	4630-34	S	-	-	-
5	4630-5	F	+	+	+	35	4630-35	F	+	+	+
6	4630-6	F	+	+	+	36	4630-36	F	+	+	+
7	4630-7	F	+	+	+	37	4630-37	F	+	+	+
8	4630-8	S	-	-	-	38	4630-38	F	+	+	+
9	4630-9	F	+	+	+	39	4630-39	S	-	-	-
10	4630-10	F	+	+	+	40	4630-40	F	+	+	+
11	4630-11	S	-	-	-	41	4630-41	F	-	-	-
12	4630-12	F	+	+	+	42	4630-42	S	-	-	-
13	4630-13	S	+	-	-	43	4630-43	F	+	+	+
14	4630-14	F	+	+	+	44	4630-44	S	-	-	-
g15	4630-15	F	+	+	+	45	4630-45	S	-	-	-
16	4630-16	S	-	-	-	46	4630-46	S	-	-	-
17	4630-17	F	+	+	+	47	4630-47	F	+	+	+
18	4630-18	F	+	+	+	48	4630-48	F	+	+	+
19	4630-19	F	+	+	+	49	4630-49	S	-	-	-
20	4630-20	F	+	+	+	50	4630-50	F	+	+	+
21	4630-21	S	-	-	-	51	4630-51	F	-	-	-
22	4630-22	F	-	-	-	52	4630-52	F	-	-	-
23	4630-23	F	-	-	-	53	4630-53	F	+	+	+
24	4630-24	F	-	-	-	54	4630-54	S	-	-	-
25	4630-25	S	-	-	-	55	4630-55	F	+	+	+
26	4630-26	F	-	-	-	56	4630-56	S	-	-	-
27	4630-27	S	-	-	-	57	4630-57	F	-	-	-
28	4630-28	F	-	-	-	58	4630-58	F	+	+	+
29	4630-29	F	-	-	-	59	4630-59	F	+	+	+
30	4630-30	S	-	-	-	60	4630-60	F	+	+	+

Notes: F: fertile; S: sterility. +: There are electrophoretic bands; -: There are no electrophoretic bands.
